# Chemical Synthesis and NMR Solution Structure of Conotoxin GXIA from *Conus geographus*

**DOI:** 10.3390/md19020060

**Published:** 2021-01-26

**Authors:** David A. Armstrong, Ai-Hua Jin, Nayara Braga Emidio, Richard J. Lewis, Paul F. Alewood, K. Johan Rosengren

**Affiliations:** 1 School of Biomedical Sciences, Faculty of Medicine, The University of Queensland, Brisbane, QLD 4072, Australia; david.armstrong@uqconnect.edu.au; 2 Institute for Molecular Bioscience, The University of Queensland, Brisbane, QLD 4072, Australia; a.jin@imb.uq.edu.au (A.-H.J.); n.bragaemidio@imb.uq.edu.au (N.B.E.); r.lewis@uq.edu.au (R.J.L.); p.alewood@imb.uq.edu.au (P.F.A.)

**Keywords:** conotoxin, venom, cone snail, inhibitor cystine knot, *Conus geographus*, disulfide-rich

## Abstract

Conotoxins are disulfide-rich peptides found in the venom of cone snails. Due to their exquisite potency and high selectivity for a wide range of voltage and ligand gated ion channels they are attractive drug leads in neuropharmacology. Recently, cone snails were found to have the capability to rapidly switch between venom types with different proteome profiles in response to predatory or defensive stimuli. A novel conotoxin, GXIA (original name G117), belonging to the I_3_-subfamily was identified as the major component of the predatory venom of piscivorous *Conus geographus*. Using 2D solution NMR spectroscopy techniques, we resolved the 3D structure for GXIA, the first structure reported for the I_3_-subfamily and framework XI family. The 32 amino acid peptide is comprised of eight cysteine residues with the resultant disulfide connectivity forming an ICK+1 motif. With a triple stranded β-sheet, the GXIA backbone shows striking similarity to several tarantula toxins targeting the voltage sensor of voltage gated potassium and sodium channels. Supported by an amphipathic surface, the structural evidence suggests that GXIA is able to embed in the membrane and bind to the voltage sensor domain of a putative ion channel target.

## 1. Introduction

The *Conus* genus is comprised of ~850 species of carnivorous marine gastropods that are commonly referred to as cone snails. There has been significant interest in cone snails due to their complex and diverse venom, which provides a rich source of both drug leads and tools in neuropharmacology [1]. Individual species are divided into three groups based on their prey preference for fish (piscivores), mollusk (molluscivores) or worm (vermivores) [2]. Conserved across the genus is a complex harpoon delivery system used to envenomate prey to induce rapid paralysis. Envenomation has also been implicated in both defensive and competitive behaviours [3,4,5].

The venom is composed of a complex cocktail of peptide toxins that individually have exquisite potency and high selectivity for a wide range of voltage- and ligand-gated ion channels [6]. The peptide toxins can be highly selective for individual sub-type members of ion channel families, a key feature that makes them desirable drug candidates with limited side effects due to off target activity [7]. Physiologically, the toxins act in synergy as groups known as cabals to elicit distinct paralytic effects. This includes the lightning cabal (rapid tetanic paralysis) and the motor cabal (neuromuscular block) [8,9]. In net hunting *C. geographus and C. tulipa,* an alternative nirvana cabal has been identified that induces sedation in prey to facilitate hunting of fish by mouth [10].

Individually, the peptide toxins are divided into two groups based on the number of disulfide bonds: conopeptides (maximum one disulfide bond) and the predominant conotoxins (two or more disulfide bonds) [11]. Conotoxins are further classified into pharmacological families based on ion channel targets and activity, as well as structural families based on their cysteine (Cys) frameworks.

In respect to complexity of venom proteome profile, a distinguishing feature of *Conus* venom is their significant inter-species diversity. It is estimated that each species produces >1000 unique toxins, with <0.1% of toxins in the genus currently being pharmacologically characterized [12,13]. Similarly, the availability of 3D structures is limited with currently only ~150 structures having been determined from >8000 native sequences [14]. While the majority of these are derived from solution NMR spectroscopy, X-ray crystallography has been utilised recently, and in particular is useful for studying large complexes with conotoxin targets [14,15]. Structure determination is an important step in developing structure activity relationships to understand conotoxin function on a molecular level. Due to their desirable pharmacological properties and natural diversity, conotoxins have been identified as potential drug leads for a range of neurological conditions. Highlighting this potential, the Ca_V_2.2 inhibitor MVIIA (Prialt^®^) has been used clinically since 2004 for the treatment of neuropathic pain [16].

An unrecognised level of sophistication to the envenomation process, was recently discovered by Dutertre et al., revealing cone snails can rapidly switch between venom types with different proteome profiles, in response to defensive or predatory stimuli [17]. Each venom type displays distinct pharmacological activity, with the defensive venom of the piscivorous *C. geographus* displaying greater activity at human ion channels than the predatory venom [17].

A prominent component of the *C. geographus’* predatory venom was the novel GXIA conotoxin, a 32 amino acid peptide with eight Cys residues. The spacing of these Cys residues (C–C–CC–CC–C–C) gives it an ‘XI’ framework, while GXIA is a member of the I superfamily and part of the I_3_ subfamily, based on its signal peptide sequence [17]. To date, the biological activity for only a few members of the I superfamily have been reported, including; κ-BtX (*C. betulinus*) an up-modulator of BK channels, ViTx (*C. virgo*) an inhibitor of K_V_1.1 and 1.3 channels, ι-RXIA (*C. radiates*) an agonist for Na_V_ 1.2, 1.6 and 1.7 channels, and Xm11a (*Conasprella ximenes*) an antimycobacterial peptide [18,19,20,21]. Based on a search of the Conoserver database and relevant literature, the only member of the I-superfamily with a reported structure found is ι-RXIA, where the majority of loops and termini differ in length, and the sequence identity to GXIA is minimal, apart from the cysteines [14,22]. Interestingly, when GXIA was isolated and injected in vivo into fish no paralytic effects were observed, leading to the hypothesis that GXIA may be part of the sedative nirvana cabal [17]. All of these factors make GXIA of significant interest and thus here we applied NMR spectroscopy to elucidate its 3D structure and identify key structural characteristics to gain new insights into its biological activity.

## 2. Results and Discussion

### 2.1. NMR Spectroscopy and Resonance Assignment

**Figure 1:**
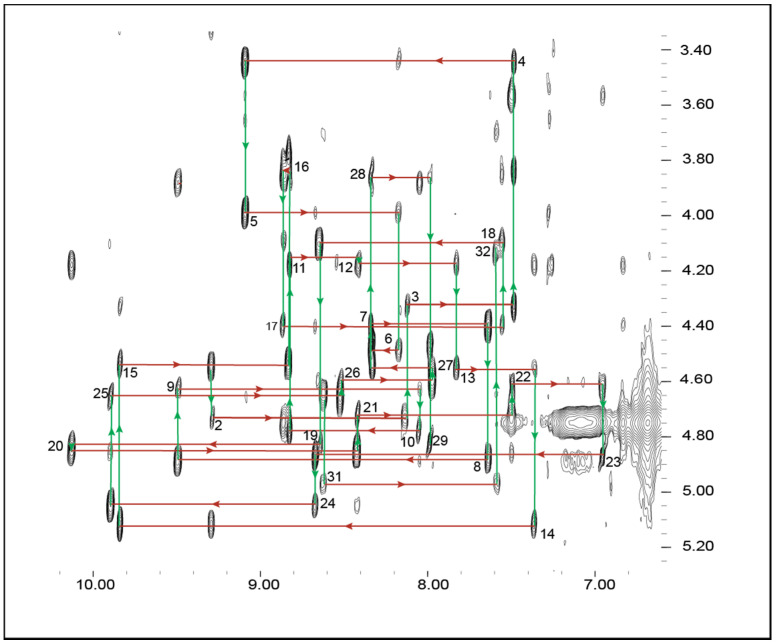
The fingerprint region of the NOESY spectrum of GXIA recorded with a mixing time of 100 ms. Residue numbers are labelled next to the intra-residue Hα_i_-HN_i_ NOE peaks identified by the sequential walk assignment strategy. Red horizontal arrows point to the inter-residual Hα_i_-HN_i+1_ NOE peaks, whilst vertical green arrows point to the intra-residual Hα_i_-HN_i_ NOE peaks.

To determine the structural features of GXIA 2D homonuclear ^1^H-^1^H NMR datasets were recorded, including total correlation spectroscopy (TOCSY) and nuclear Overhauser effect spectroscopy (NOESY) spectra at 600 MHz on a sample containing 1 mg/mL of GXIA. All spectra were of high quality with minimal signal overlap as a result of excellent dispersion and sharp resonance signals, indicating a highly structured peptide (Figure 1) [23]. Complete resonance assignments were achieved through sequential assignment strategies [24]. GXIA contains one proline residue (Pro30), for which strong Hα_i_-Hδ_i+1_ NOESY peaks indicate a *trans* conformation of the X-Pro bond.

In addition to ^1^H homonuclear data 2D ^1^H-^13^C and ^1^H-^15^N heteronuclear single-quantum correlation spectroscopy (HSQC), data were recorded at natural abundance, utilising the excellent sensitivity and signal-to-noise of the cryoprobe. ^13^C and ^15^N assignments could be inferred from the proton shifts and helped to confirm side chain assignments given the characteristic ^13^C shifts of various amino acids. The ^13^C and ^15^N chemical shifts were also useful for determining dihedral angle restraints using TALOS-N [25].

### 2.2. Disulfide Connectivity

There are eight Cys residues in GXIA, and their disulfide connectivity was determined in two stages. First by analysis of cross peaks in the NOESY (100 ms mixing time) spectrum, which indicated the Cys residues’ proximity to each other. Cysteines 14–15 and 19–20 showed strong cross peaks in the NOESY spectrum. However, vicinal disulfide bridges are extremely rare and it was assumed these cross peaks were solely due to the residues’ close proximity as sequential neighbours. Initially, Hα 20 to HN 8, reinforced by Hβ_3_ 20 to Hβ_2_ 8 cross peaks were identified. Distinct Hβ_2&3_ 1 to HN 15 peaks, and Hβ_2_ 19 to Hβ_3_ 31 peaks were also observed. For the final two Cys residues, cross peaks between Hβ_2_ 14 to Hβ_3_ 24 were identified. Therefore, the predicted disulfide connectivity was Cys1-Cys15, Cys8-Cys20, Cys14-Cys24 and Cys19-Cys31 (I-IV, II-VI, III-VII, V-VIII). This connectivity is consistent with an ICK+1 motif, where Cys1-Cys15, Cys8-Cys20, and Cys14-Cys24 form the cystine knot, while the additional disulfide bond is Cys19-Cys31. This disulfide connectivity is similar to the one reported for ι-RXIA, the only other structure from the I-superfamily [22].

Inclusion of the Cys side chain conformations as restraints during structure calculations has been previously shown to be a powerful way of confirming correct disulfide connectivity [26]. Cys side chain dihedral angles (χ^1^) were resolved by analysis of inter proton HN-Hβ NOESY intensities, in conjunction with ^3^J_HαHβ_ coupling constants obtained from an exclusive correlation spectroscopy (E.COSY) spectrum. For Cys residues 14, 15, 19 and 24 one small and one large ^3^J_HαHβ_ coupling constant were observed, together with one weak and one strong HN-Hβ NOE peak, indicating a χ^1^ dihedral angle of −60°. For the remaining Cys 8 and 20, again one small and one large ^3^J_HαHβ_ coupling constant were observed, but the HN-Hβ NOE peaks were of equal intensity, confirming a χ^1^ dihedral angle of 180° for these residues. Ultimately, the disulfide connectivity was confirmed by structure calculations using both distance and dihedral restraints.

With a known connectivity framework, we also used the program DISH to predict χ^1^ and χ^2^ angles based on chemical shift inputs [27]. GXIA has an N-terminal Cys residue that was excluded from DISH predictions. The remaining seven χ^1^ angles predicted from DISH were in full agreement with those derived from the NOESY and E.COSY data, reassuring that the deduced connectivity was correct. DISH has a high degree of accuracy for the prediction of χ^2^ angles when the χ^1^ angle is known [27]. Therefore, all Cys χ^1^ and χ^2^ angles could be included as restraints in the final rounds of simulated annealing of structures in CNS.

### 2.3. Secondary Hα Shifts

**Figure 2:**
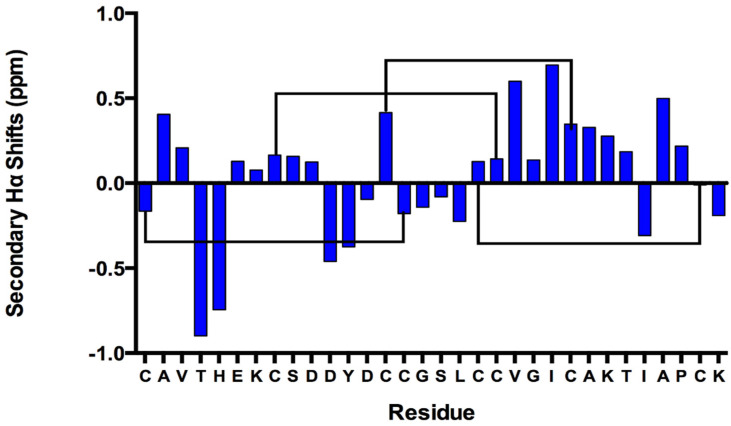
The secondary Hα shifts (ppm) for all residues of the GXIA conotoxin, listed with sequence on the *x*-axis. Stretches of positive values indicate the presence of β-strands, whereas stretches of negative values indicate turns and helical structures. Black bars represent the proposed disulfide bond connectivity between Cys residues.

Secondary Hα shifts were calculated as the difference between the observed GXIA Hα shifts and the random coil (RC) Hα shifts (Figure 2) [28]. Stretches of positive secondary Hα secondary shifts between residues 6–10 and 19–27 indicate the presence of a β-strand secondary structure [29]. Residues 19–27 could either form a long singular β-strand or, more likely given the restraints of the four disulfides, two strands connected by a tight turn forming a β-hairpin [29].

### 2.4. Backbone Amide Hydrogen Bonds

**Figure 3:**
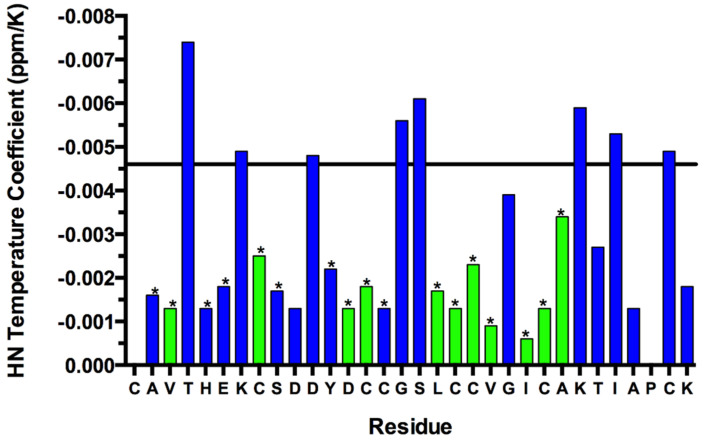
Backbone amide proton temperature coefficients of GXIA residues. Residue 1 is not visible due to fast exchange of the free amine, whilst residue 30 is a proline. Amide protons with a temperature coefficient > −0.0046 ppm/K (represented by horizontal black line) are likely to be involved in hydrogen bonding. Residues with backbone amide protons observed to be slow exchanging in deuterated solvent are shown in green. Residues marked with a * had confirmed backbone amide hydrogen bonds included in calculations.

**Figure 4:**
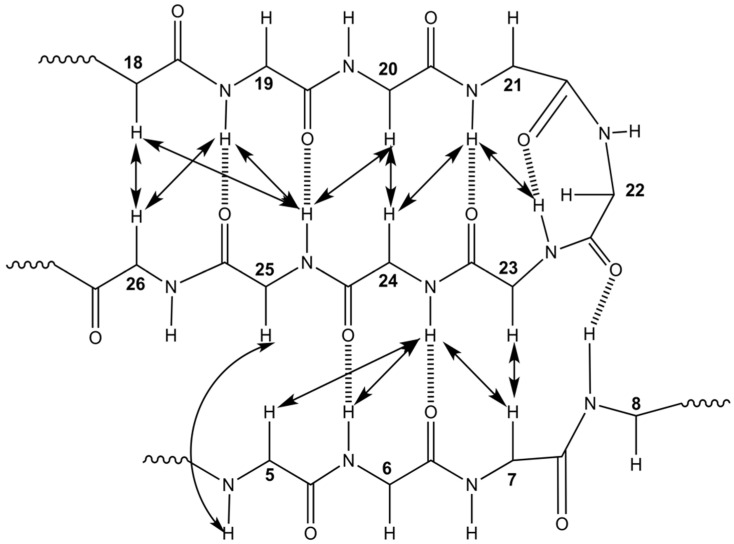
A schematic drawing of the predicted triple-stranded antiparallel β-sheet of GXIA formed by strand I (residues 6–8), strand II (18–21) and Strand III (23–26). Double arrowed lines represent inter-residual NOE cross peaks for non-neighbouring amide protons, whereas dashed lines represent hydrogen bonds.

Both temperature coefficients (TC) (Δδ_HN_/ΔT) and deuterium (D_2_O) exchange data were used to analyse the presence of hydrogen bonds. Backbone amide protons with a TC > −0.0046 ppm/K and/or observed to be slow exchanging in D_2_O are considered likely to be hydrogen bonded (Figure 3) [30,31]. Hydrogen bond acceptors were subsequently identified by structure calculations for all slow exchanging amides, and most amides with TC suggesting involvement in hydrogen bonds. Based on the RC values, observed NOE peaks between non-neighbouring residues and predicted hydrogen bonds, we concluded the central feature of GXIA is a triple-stranded anti-parallel β-sheet (Figure 4). Strand I consists of residues Glu6-Cys8, strand II of Leu18-Cys21 and strand III of Cys23–Lys26. The observed NOE connectivity and predicted hydrogen bonds for the β-strands are shown in Figure 4.

### 2.5. Structure Determination

For the full 3D structure determination of GXIA, structural restraints were collected from the NMR data. These included 41 backbone dihedral angles based on a TALOS-N analysis of ^1^H, ^13^C and ^15^N chemical shifts, and 21 side chain dihedral angles established by analysis of coupling constants, NOE patterns, TALOS-N, and DISH [25,27,32]. Additionally, cross peaks in the 100 ms NOESY were selected and integrated, then assigned automatically to inter proton distances during the simulated annealing protocol within the program CYANA [33]. This resulted in 380 unambiguous distance restraints. Finally, 34 hydrogen bond restraints were added based on the identification of 17 hydrogen bond. In the final round of calculations, 50 structures were calculated using a simulated annealing procedure and refinement in explicit water using the program CNS [34]. The 20 structures with the lowest energies, as well as good covalent geometry and stereochemical quality based on MolProbity scores, were selected to represent the structure of GXIA. Their structural and energy statistics are presented in Table 1. These structures are well defined and in excellent agreement with experimental data with no NOE distance violation greater than 0.2 Å, no dihedral angle violation greater than 2° and a backbone pairwise RMSD of 0.60 Å (Table 1).

### 2.6. GXIA Structural Features

**Figure 5:**
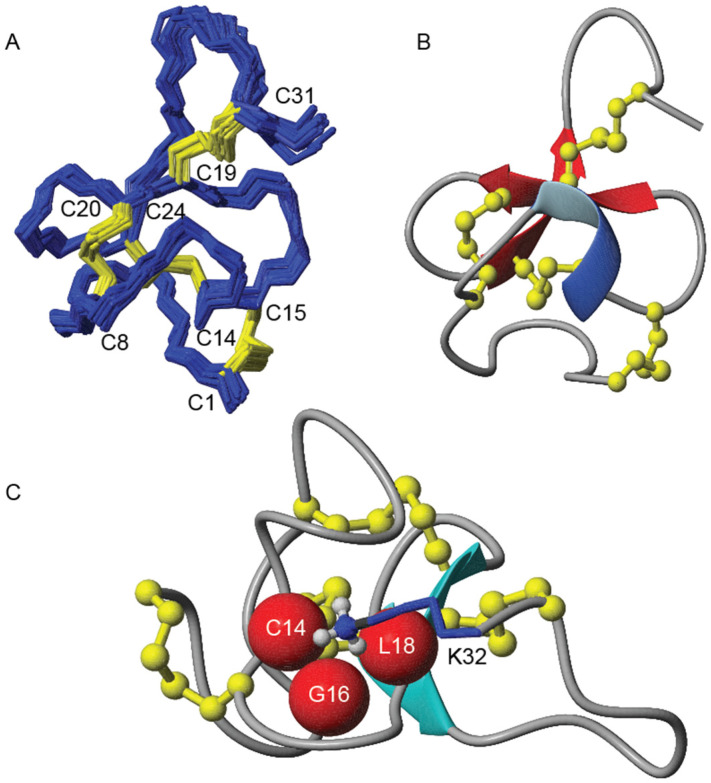
Solution structure of GXIA calculated using CNS and visualised using MolMol (**A**) The backbone of the 20 lowest energy structures superimposed on each other, with disulfide bonds shown in yellow. (**B**) The lowest energy structure in ribbon representation. β-strands as arrows, helical turns as ribbons and disulfide bonds as ball-and-sticks. (**C**) The lowest energy structure in ribbon form showing the Lys32 folding in towards the centre of the protein due to interactions with the Cys14, Leu18 and Gly16 residues. The short strand comprising residues 6-8 is not formally recognized as part of the β-sheet by MolMol.

The structure of GXIA was analysed using MolMol (Figure 5) [35]. As predicted based on secondary Hα shifts, the peptide is highly constrained, including at both termini as seen by the overlay of the of structural family (Figure 5A). This is partly due to the presence of Cys residues involved in disulfide bonds at, or close to, the termini (Cys1 and Cys31). The disulfide bonds do form an ICK+1 motif, which is also found in other conotoxin peptides [36]. Figure 5B shows the lowest energy structure in ribbon representation. The two anti-parallel β-strands of the hairpin that was predicted based on observed NOE interactions and secondary Hα shifts (Figure 3) are evident, while the third strand involving residues 6–8 is more irregular, and not formally recognised as part of the sheet by the software. An additional secondary motif observed is a 3_10_ helical turn formed by residues Asp10–Asp13. The C-terminus has an unusual feature; the positively charged Lys32 side chain, which would be expected to be solvent exposed, extends towards the centre of the protein forming electrostatic interactions with the carbonyls of Cys14, Gly16 and Leu18 (Figure 5C). This arrangement was confirmed by the narrow line width of the Lys32 NH_3_ resonance, which is normally very broad due to exchange with solvent.

### 2.7. Structural Comparison

**Figure 6:**
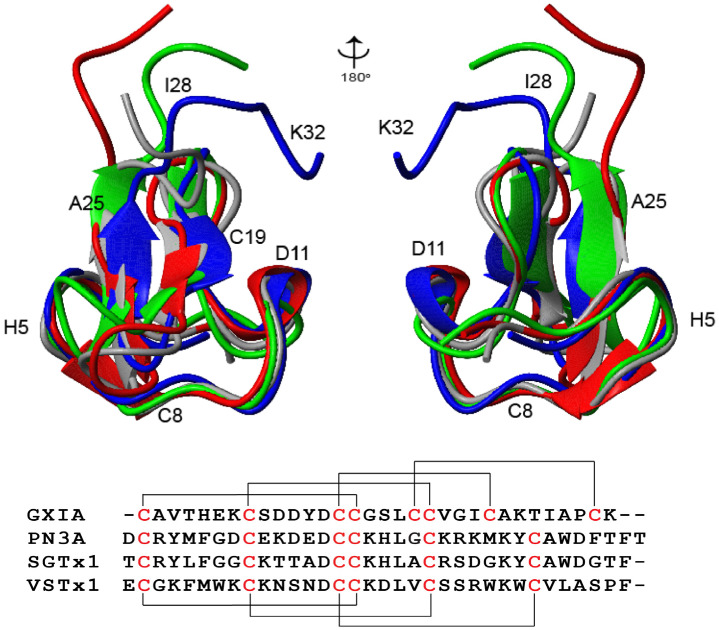
A Ribbon view showing secondary structure of GXIA (blue), VSTx1 (green), Pn3a (grey) and SGTx1 (red) overlaid on each other. The two figures show a rotation around the *Y*-axis of 180°. Bottom panel shows an alignment of the primary sequence of GXIA and the spider toxins. Cysteine residues are shown in red. Black bars on the top indicate disulfide bonding for GXIA, whilst those below indicate disulfides for VSTx1, Pn3A and SGTx1. The short N-terminal strand is only formally recognized as part of the β-sheet by MolMol in the structure of SGTx1, but conformationally is very similar in all structures.

The novel conotoxin GXIA was identified as the predominant peptide of *C. geographus*’ predatory venom, though surprisingly it elicited no paralytic effects when injected into fish during in vivo studies [17]. GXIA belongs to the I_3_ superfamily, for which no structures or pharmacological activity has been reported to date; hence it was of interest to characterize this peptide further. Having determined the structure using solution NMR spectroscopy, allowed us to search for other peptides in the Protein Data Bank with 3D structural conservation to GXIA using the Dali server [37]. Interestingly, the top three matches were all spider toxins, Pn3a (PDB 5t4r) from *Pamphobeteus nigricolor*, VSTx1 from the *Grammostola spatula* (PDB 1s6x) and SGTx1, isolated from *Scorda grisipes* (PDB 1la4) [38,39,40,41,42]. Pn3a is a selective voltage gated sodium channel (Na_V_) 1.7 inhibitor [42], whilst VSTx1 and SGTx1 are members of the voltage gated potassium (K_V_) voltage sensor (VS) toxin family. All three toxins modulate their targets by binding to the VS domain [40,43,44,45]. GXIA displays a highly conserved backbone structure relative to all three toxins, including the secondary structural motifs of two central anti-parallel β-strands, a helical turn and several loops (Figure 6). In SGTx1 the extra β-strand at residues Gly7-Cys9 is more regular and formally recognised by MolMol [40,41,46]. The toxins all have multiple disulfide bonds like GXIA and the formation of an ICK is conserved across all toxins of the family [40,41,44]. However, the extra disulfide of the ICK+1 motif and the unusual conformation of the Lys32 side chain result in GXIA having a more constrained C-terminus, and overall a more compact structure compared to the other toxins (Figure 6).

Analysis of the primary sequence shows that only seven residues are conserved across all four structures (Figure 6). The side chains of the two conserved non-Cys residues in GXIA, Asp13 and Leu18, adopt relatively buried positions within the peptide. This suggests that they contribute to the structure, rather than peptide interactions.

### 2.8. Electrostatic Surface and Proposed Activity as a Voltage Sensor Toxin

VSTx1 and SGTx1 are a part of the K_V_ VS toxin family, members of which modulate K_V_ channel activity by binding to the VS domain [40,43,44,47]. The toxins partition into the cellular membrane and bind to the S3b and S4 helices of the VS domain, stabilising the resting state, hence increasing the energy required for K_V_ channel activation [40,41,43,48,49,50]. A key conserved structural motif of all K_V_ VS toxins is their amphipathic nature, which allows them to insert into the membrane and access binding sites of the S3b and S4 helices located near the extracellular surface [40,41,43,44,47,48,51,52]. Pn3A also displays an amphipathic surface and binds to the S3 and S4 domains and alters voltage dependence for Na_v_1.7 activation [42].

**Figure 7:**
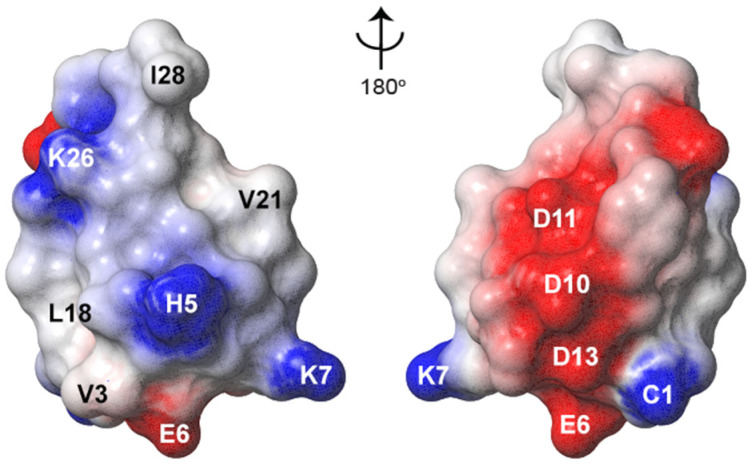
Electrostatic surface of GXIA calculated in Molmol. Neutral residues are in grey, basic residues are in blue and acidic in red. The two figures are rotated around the *Y*-axis 180°.

Based on the conserved amphipathic nature of VS toxins, the electrostatic surface of GXIA was analysed (Figure 7). It has an acidic surface patch consisting of Glu6, Asp10, Asp11 and Asp13. A hydrophobic face is also present, but punctuated by the basic His5 residue. The histidine side chain has a pKa value of 6.10, therefore at physiological pH the majority of histidine residues will have a neutral charge. Hence, GXIA can be considered as displaying a hydrophobic face composed of residues Val3, Leu18, Val21, Ile23 and Ile28 (Figure 7).

Based on the conservation of the peptide backbone and amphipathic nature of GXIA to known VS toxins, we hypothesized that GXIA modulates ion channels by binding to the VS domains. GXIA has no paralytic effects when injected into fish [17], thus it was suggested by Dutertre et al. (2014) that the GXIA biological function may be part of the sedative nirvana cabal which suppresses electrical activity [12,17]. K_V_ VS toxins do not completely inhibit K_V_ channel activation, but rather result in a more positive action potential, and an increased depolarization can still cause channel activation [47,49,50]. Therefore, the proposed activity of GXIA is consistent with its observed non-paralytic effect. Whilst another I-superfamily member, ViTx1, has been reported to inhibit K_V_ activity, based on theoretical modelling it is suggestive that ViTx1 acts as a pore blocker [19,53]. The obvious future direction will be to test the proposed biological activity of GXIA, as well as ion channel specificity and analyse whether the structural conservation to K_V_ VS toxins translates into conserved pharmacological action.

### 2.9. Side Chain Positions

**Figure 8:**
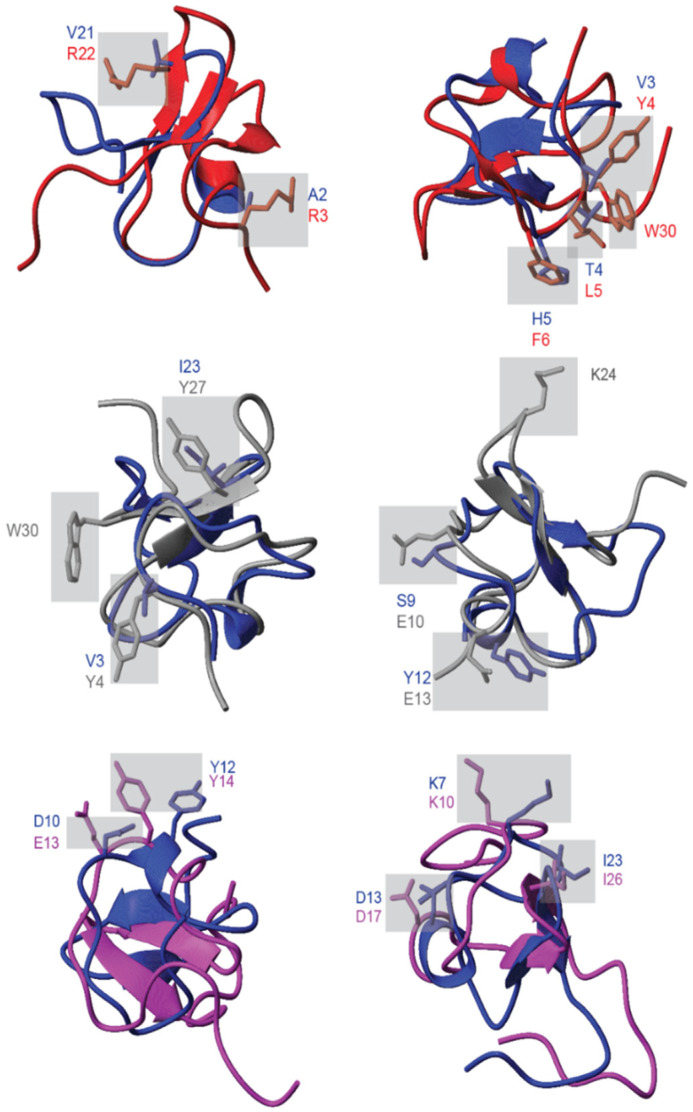
Comparison of side chain positions of GXIA (blue) to SGTx1 (red), Pn3a (grey) and ι-RXIA (purple). Residues that are known or hypothesized to be functionally important for SGTx1 and Pn3a are highlighted in grey. If present a comparable side chain for GXIA is also shown. For ι-RXIA side chains identified as being conserved are highlighted. The ι-RXIA and GXIA backbones were aligned based on these residues.

An alanine (Ala) scan conducted on SGTx1 identified Arg3, Tyr4, Leu5, Phe6, Arg22 and Trp30 as key residues essential for activity. For Pn3a, an extensive structural analysis comparing it to other selective Na_v_1.7 toxin inhibitors showed Tyr4, Glu10, Glu13, Lys24 and Trp30 as residues important for activity. The positions of these key side chains in SGTx1 and Pn3a were compared to GXIA to see if analogous residues were present in these spatial regions. Minimal conservation of these side chains was observed (Figure 8). This indicates the structural conservation is predominantly limited to the backbone, and differences in the nature of the side chains may suggest a different biological activity for GXIA. The only other structure of a conotoxin with a Cys XI framework is ι-RXIA, which has little sequence conservation to GXIA. ι-RXIA is an agonist for Na_V_ 1.2 1.6 and 1.7 channels, shifting the voltage dependence activation of the channels to a more hyperpolarized activation state based on both rodent channels in Xenopus oocytes and mouse neurons [20]. Interestingly, despite these divergences in the sequences, the backbone conformation of ι-RXIA and GXIA are similar with the same secondary structure motifs. Comparing side chains in the same spatial region, a number of positions and biochemical properties also appear to be conserved, unlike the case of SGTx1 and Pn3a. This includes a number of charged residues and structural motifs such as the hydrophobic Tyr followed by an acidic residue (Figure 8). No mutational studies have been performed to confirm the importance of these residues to the overall activity of ι-RXIA; however, the degree of conservation of residues may be indicative of conservation for a shared target.

## 3. Experimental Section

### 3.1. Peptide Synthesis and Co-Elution

Linear GXIA was synthesized by standard manual Fmoc-chemistry starting from Fmoc-Lys(Boc)-Wang resin on a 0.6 mmol scale. The deprotection of the side chain group and cleavage of the linear peptide from the resin were conducted with a TFA (87.5%): H_2_O (5%): TIPSi (5%): EDT (2.5%) solution for 3 h. Then the solution was filtered to remove the resin and the peptide precipitated with cold ether. The precipitate was dissolved in 50% acetonitrile/0.05% TFA/H_2_O, filtered and lyophilized on a freeze dryer. The crude peptide was purified by preparative RP-HPLC (Vydac C18). The fractions collected were analysed by ESI-MS and analytical HPLC was performed on the fractions with the desired mass to evaluate purity. The pure fractions were combined and lyophilized. Random oxidation was achieved in 0.33 M NH_4_OAc/0.5 M GnHCl at pH 6.7 with 1:100:10 peptide:GSH:GSSH at a peptide concentration of 0.1 mg/mL. The oxidation was performed at 4 °C for 72 h before subsequent purification of the oxidized peptide by HPLC.

To confirm the native disulfide arrangement co-elution was carried out on an ABSCIEX QSTAR Pulsar mass spectrometer. A predatory venom and the synthetic peptide were subjected to LC-ESI-MS at 1% B/min gradient. The LC separation was achieved using a Thermo C18 4.6 × 150 mm column. Extracted ion chromatographs were compared and confirmed the right material.

### 3.2. NMR Spectroscopy

All NMR spectroscopy experiments were performed on a Bruker Avance 600 MHz spectrometer equipped with a cryoprobe. Samples for NMR spectroscopy were prepared by dissolving 0.5 mg of peptide in 0.5 mL of either 90% H_2_O/10% deuterium oxide (D_2_O) or 100% D_2_O at pH 5.1.

A series of 2D homonuclear spectra were recorded at 298 K. This included TOCSY, NOESY, E.COSY, ^1^H–^13^C HSQC and ^1^H–^15^N HSQC spectra. The homonuclear ^1^H-^1^H experiments were recorded over a sweep width of 12 ppm with 4096 data points in the direct dimension and 512 increments in the indirect dimension, which was zero-filled to 1024 points prior to Fourier transformation. The heteronuclear ^1^H-^15^N HSQC experiment was recorded at natural abundance of ^15^N (<0.5%) using 256 scans, with resolutions of 2048 points over 12 ppm in the direct dimension and 128 points (Linear predicted to 256 and zero-filled to 512) over 35 ppm in the indirect dimension. The heteronuclear ^1^H-^13^C HSQC experiment was recorded at natural abundance of ^13^C (~1%) using 128 scans, with resolutions of 2048 points over 12 ppm in the direct dimension and 256 points (Linear predicted to 512 and zero-filled to 1048) over 100 ppm in the indirect dimension. TOCSY mixing time was 80 ms, while NOESY mixing time was 100 ms. Chemical shifts were referenced to 4,4-dimethyl-4-silapentane-1-sulfonic acid (DSS) at 0.0 ppm [28].

CARA was used for cross peak assignment and integration, whilst distance restraints were derived from cross peak intensities by the program CYANA [33,54]. Additionally, CYANA was used for automatic assignment of NOE cross-peaks based on the determined chemical shifts and analysis of initial structures [33].

^13^C and ^15^N HSQC spectra were assigned using ^1^H chemical shifts from the homonuclear data. Hα, HN, Cα, Cβ and N chemical shifts were used to derive backbone dihedral angle (ϕ and ψ) restraints using TALOS-N [25]. The side chain dihedral angle (χ^1^) predictions made by TALOS-N were further confirmed by ^3^J_Hα-Hβ_ coupling constants via the E.COSY spectrum. The Hβ stereospecific conformations were determined by joint analysis of ^3^J_Hα-Hβ_ coupling constants, as well as the pattern and intensities of HN-Hβ NOESY peaks [32]. Cysteine (χ^1^ and χ^2^) side chain angles were determined using DISH [27].

Backbone amide hydrogen bond restrains were also obtained by calculating temperature coefficients (TC) as described by Anderson et al. (1997) from the chemical shifts observed in multiple ^1^H TOCSY spectra obtained at varying temperatures between 288–308 K. This was supported by analysis of the TOCSY spectra recorded for a fully protonated peptide after it was dissolved in 100% D_2_O at 298 K. The presence of cross peaks from amide protons in spectra recorded several hours after the peptide was dissolved in 100% D_2_O indicated that these were protected from the solvent by the presence of hydrogen bonds.

Initial structures were calculated using torsion angle molecular dynamics by the program CYANA, and the final set of structures were calculated and refined in explicit water using CNS [33,34]. The best 20 structures were visualised using MOLMOL, whilst MOLPROBITY was used to assess stereochemical quality of the final models [35,55]. Final structures were compared to known structures in the PDB via the Dali server [37]. The structure, restraints and chemical shifts have been submitted to the PDB and BMRB and given the access codes 6CEI and 30406, respectively.

## 4. Conclusions

GXIA is the first reported structure for a conotoxin belonging to the I_3_ subfamily, and only the second structure after ι-RXIA from the I-superfamily, which currently contains ~130 sequences. The 3D structure was resolved using 2D NMR, with the 20 final structures selected to represent the peptide showing excellent agreement with experimental data. GXIA is characterized by a triple stranded anti-parallel β-sheet, with eight Cys residues forming an ICK+1 motif. It is apparent that the backbone for GXIA adopts a common scaffold conserved across multiple toxins, from different species that have very divergent activities. The difference in selectivity at K_V_ and Na_V_ channels as well as agonist or antagonist behavior appears to be dictated by differences in the side chains. While the activity of GXIA is unknown, it appears to have a high degree of side chain conservation with ι-RXIA, a Na_V_ agonist. The fact that the ICK toxin family appears capable of extensive chemical modification that change pharmacological activity is an interesting perspective, and suggests that these toxins may be manipulated for tuning specific activities. While the obvious targets for GXIA are K_V_ and Na_V_ channels, its functional role in prey capture is less clear, suggesting it may act in synergy with other conotoxins. Future elucidation of activity will determine if GXIA is of therapeutic relevance and allow for the development of more refined structure-activity relationships to further understand the conotoxin I_3_-superfamily. The availability of the GXIA structure will also allow modelling of other members and rational assessment of conserved features that can be explored through mutational studies.

## Figures and Tables

**Table 1:** Energy and structural statistics of the 20 lowest energy structures of GXIA calculated using a simulated annealing procedure within CNS.

**Energies:**	
Overall (kcal/mol)	−1185 ± 20.1
Bonds (kcal/mol)	10.6 ± 0.81
Angles (kcal/mol)	33.6 ± 2.2
Improper (kcal/mol)	13.3 ± 1.7
Dihedral (kcal/mol)	145 ± 1.1
Van Der Waal (kcal/mol)	−126.2 ± 3.9
NOE (kcal/mol)	0.013 ± 0.0049
cDih (kcal/mol)	0.32 ± 0.21
Electrostatic	−1261 ± 21.8
**RMS:**	
Bonds (Å)	0.0100 ± 0.00041
Angles (°)	1.01 ± 0.034
Improper (Å)	1.43 ± 1.1
Dihedral (Å)	41.3 ± 1.6
NOE (Å)	0.0054 ± 0.0011
cDih (Å)	0.20 ± 0.064
**MolProbity Analysis:**	
Clashscore	9.43 ± 2.22
Poor Rotamers	0 ± 0
Ramachandran Favoured	88.8 ± 2.5%
Ramachandran Outliers	0 ± 0
Molprobity Score (% rank)	2.06 ± 0.14 (72 ± 7.3%)
**Pairwise RMSD:**	
Backbone atoms (Å)	0.60 ± 0.18
Heavy atoms (Å)	1.07 ± 0.19
**Experimental data:**	
NOE distance restraints:	
Sequential (i–j = 1)	112
Medium range (i–j < 5)	50
Long range (i–j ≥ 5)	99
Dihedral restraints:	41 backbone/21 side chain
Hydrogen bond restraints:	34 (for 17 hydrogen bonds)
NOE violations > 0.2 Å	0
cDih violations > 2.0°	0
